# Plagues, Public Health, and Politics^1^

**DOI:** 10.3201/eid1011.040673

**Published:** 2004-11

**Authors:** Jeffrey P. Koplan, Melissa McPheeters

**Affiliations:** * Emory University, Atlanta, Georgia, USA

**Keywords:** public health, health policy, infectious disease

Dr. Koplan (Figure 2) is vice president for academic health affairs at Emory University. His research interests include the spectrum of public health disciplines.

**Figure 2:**
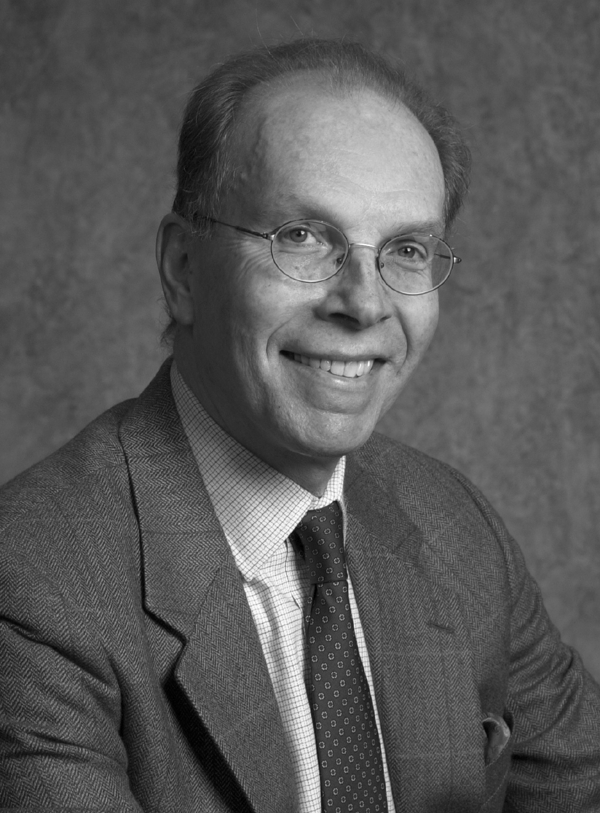
Dr. Koplan is vice president for academic health affairs at Emory University. His research interests include the spectrum of public health disciplines.

Dr. McPheeters (Figure 3) is a healthcare epidemiologist whose work focuses on translation and use of research in policy and practice, particularly in the areas of women’s health and pregnancy care.

**Figure 3:**
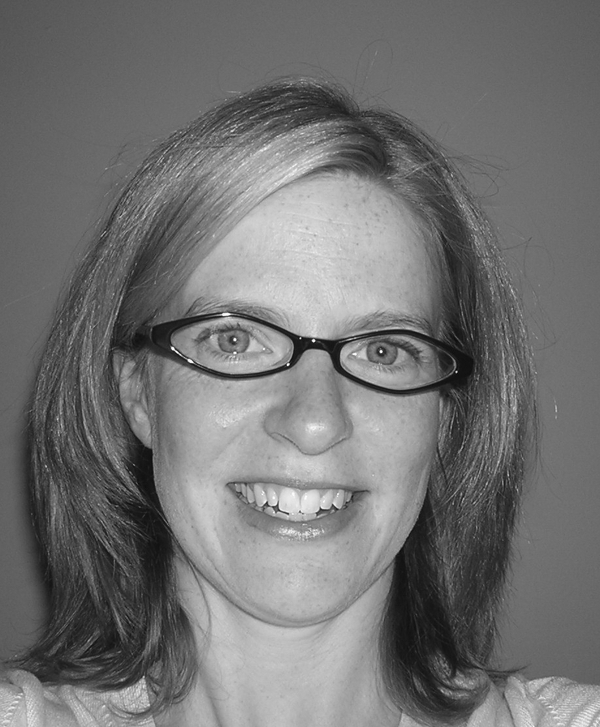
Dr. McPheeters is a healthcare epidemiologist whose work focuses on translation and use of research in policy and practice, particularly in the areas of women’s health and pregnancy care.

On July 1, 1665, the lord mayor and aldermen of the city of London put into place a set of orders "concerning the infection of the plague," which was then sweeping through the population. He intended that these actions would be "very expedient for preventing and avoiding of infection of sickness" (*1*).

At that time, London faced a public health crisis, with an inadequate scientific base in that the role of rats and their fleas in disease transmission was unknown. Nonetheless, this crisis was faced with good intentions by the top medical and political figures of the community.

Daniel Defoe made an observation that could apply to many public health interventions then and today, "This shutting up of houses was at first counted a very cruel and unchristian method… but it was a public good that justified a private mischief" (*1*). Then, just as today, a complex relationship existed between the science of public health and the practice of public health and politics. We address the relationship between science, public health, and politics, with a particular emphasis on infectious diseases.

Science, public health, and politics are not only compatible, but all three are necessary to improve the public's health. The progress of each area of public health is related to the strength of the other areas. The effect of politics in public health becomes dangerous when policy is dictated by ideology. Policy is also threatened when it is solely determined by science, devoid of considerations of social condition, culture, economics, and public will.

When using the word "politics," we refer not simply to partisan politics but to the broader set of policies and systems. Although ideology is used in many different ways, in this case, it refers to individual systems of belief that may color a person's attitudes and actions and that are not necessarily based on scientific evidence (*2*).

## Public Health Achievements

Science influences public health decisions and conclusions, and politics delivers its programs and messages. This pattern is obvious in many of public health's greatest triumphs of the 20th century, 10 of which were chronicled in 1999 by the Centers for Disease Control and Prevention (CDC) as great public health achievements, and several of which are presented below as examples of policy affecting successes (*3*). These achievements remind us of what can be accomplished when innovation, persistence, and luck converge, along with political will and public policy.

## Vaccination

Childhood vaccinations have largely eliminated once-common, terrible diseases, such as polio, diphtheria, measles, mumps, and pertussis (*4*). Polio is being eradicated worldwide. The current collaboration between the World Health Organization, the United Nations Children's Fund, CDC, and Rotary International is a political as well as biological "tour de force," and eradication of polio in Nigeria has been threatened by local political struggles and decisions. In the United States, politics has contributed to successful public health policies by requiring vaccination at school entry, which has been vital to achieving high vaccine coverage in young children.

Debate about vaccines offers an example of the effect of ideology on public health progress in the form of persons who oppose vaccination. These persons put communities at risk by refusing vaccination for themselves and their children and enlist political support to undermine our greatest medical advance.

## Family Planning

Safe contraception and family planning have not only improved the health of women by preventing unintended pregnancies, but they have also contributed to one of the century's most dramatic social revolutions by helping redefine roles and opportunities for women (*5*). However, ideologic views on contraceptive practices and sexually transmitted disease (STD) prevention continue to contradict scientific observations, which leads to compromised public health policies.

## Control of Infectious Diseases

Clean water, treated to protect us from outbreaks of infections like cryptosporidiosis, is an obvious example of the interaction between public policy and infectious disease control. Public policy has sought to control infectious disease throughout history, including attempts to ban spitting in the streets around the turn of the century (an issue that resurfaced 100 years later in the context of severe acute respiratory syndrome [SARS]) and imposing restaurant inspections to ensure sanitary conditions in food preparation. Many important infectious disease issues have political and economic overtones: Legionnaires' disease and hotel closures, Nipah virus outbreaks and the swine industry, hantavirus and the cultural and political interplay with Native American communities, and drug resistance and inappropriate and widespread antimicrobial drug use in the food industry and medicine are just a few examples (*6*).

## Recognizing Tobacco Use as a Health Hazard

Knowing that tobacco is addictive and dangerous alone did not ensure that tobacco companies were held responsible for their role in impairing many people's health. Rather, that accomplishment required a combination of political will and social insistence (*7*). Nonetheless, regulations on secondhand smoke continue to be debated, as science and individual ideology clash. These clashes become especially acrimonious as they reflect culture around a native-grown substance and often the product on which a state's economy has depended.

## New Challenges

In just the past 2 years, new health challenges have occurred that illustrate the tension between economic health of a community or a business and the personal health of citizens or employees, for example, or the role of the individual versus the government in taking responsibility for health and health-related actions. In emerging infectious diseases, these new health challenges include avian flu and bovine spongiform encephalopathy, as well as SARS.

What makes infectious diseases particularly compelling to the public, to public health and political involvement, is that microbial agents are frightening. They come from exotic places, jump from person to person, often have no treatments or preventive measures available, and can paralyze industries and communities. Infectious agents represent our lack of control over our health, regardless of whether they are used deliberately by terrorists or are delivered by nature. Many infectious diseases have become a security issue, bringing a new set of "partners" to the microbiologic and public health table. While this arrangement is appropriate and necessary in many instances, it also has potential for abuse, by promoting anxiety and insecurity for political means, distorting public health priorities, and possibly militarizing public health institutions.

## Smallpox

The decision to implement widespread vaccination against smallpox generated substantial interest in the general public. After believing that smallpox was not a threat for many years, we were informed by the government that cause for serious alarm existed.

Production of large quantities of vaccine was accelerated, which was a prudent and decisive action. This action was followed by a policy that called for vaccinations for hundreds of thousands of healthcare workers and millions of first responders. The science on which this decision was based seemed shaky at best, and many chose to forego vaccination, including two distinguished academic infectious disease units. The Washington Post criticized these units, saying, "There are reasons, moral and medical, to deplore the decision of those doctors who refuse in this manner…. Their job is not to assess intelligence risks or to second-guess state public health officials but to be prepared to care for sick people, and to vaccinate healthy people" (*8*).

The Post's statement may be correct, but academic infectious disease specialists have every right and responsibility to question decision-making that affects their patients and colleagues, especially when the scientific-political interface regarding that decision is unclear. Careful review of the literature and expert experience predicted substantial risks from adverse vaccination reactions.

The Washington Post editors seem to have missed the concept of "do no harm." Analytic and compassionate physicians realized that, in the face of little or no threat of an attack, widespread use of a potentially toxic vaccine was not in the best interest of their patients. The decision by various academic medical centers not to widely vaccinate hospital and medical personnel seems prudent, given the revised estimates of risk and the reporting of substantial adverse reactions.

Bioterrorism is not the only infectious disease challenge with political implications. Existing pathogens and newly emerging diseases remind us that infectious agents can destabilize our social structure and commerce, and they may require political or policy intervention. Therefore, the danger is that ideologic stances may intrude on the process and push us away from science and even away from good public health practice.

## SARS

The SARS outbreak in Asia in 2003 provided examples of how ideology and politics can interfere with public health practices and bring criticism by ideologues. Moreover, SARS demonstrated the challenge of protecting the public's health across national and ideologic lines. The SARS outbreak was not reported by the Chinese government for the first several months of its transmission (*9*). An ideologic perspective that required not sharing weaknesses or inadequacies with the rest of the world probably played a role in this delay. The political pressure of the rest of the world was required to convince China to acknowledge the problem and accept help.

Hong Kong, on the other hand, was more open. Early cases of atypical pneumonia were identified and reported. Further cases were ascertained, and contact tracing was put in place. The system responded with infection control efforts, including isolation and quarantine. Nonetheless, Hong Kong faced a daunting task, with a high population density and a poorly understood disease. In the end, Hong Kong's department of health faced substantial criticism from political opposition and the press, and a committee was formed to evaluate their response. The committee developed a number of recommendations but recognized overall the impressive response of the hardworking public health and healthcare communities (*10*). Nonetheless, persons initially critical of the response itself took the opportunity to criticize the report by an international panel. Certainly, being critical and trying to improve performance are valuable, but are they best done in the middle of the challenge and with blatant political intent?

## 2003–2004 Flu Season

For influenza, the scientific and political processes need to be improved. For many years in public health, we have recognized the threat of pandemic flu and called for the need to act (*11*). In this case, politics is more than helpful, it is essential. Preventing a flu pandemic necessitates using the resources of science, politics, and the private sector. Last year, vaccine development became a matter of public concern when several children died from influenza early in the season, and the press reported that the vaccine may have lacked protection against the circulating Fujian strain.

Public discussions highlighted the imperfections of science, particularly related to vaccine production and distribution. Then the finding of cases of H5N1 influenza in Asian chicken flocks and other birds and several human infections and deaths rekindled apprehension about a flu pandemic with a new, lethal strain, should mutations permit person-to-person transmission. With avian flu, some government officials were slow to disclose infected flocks to protect economic interests, and these decisions could have had tremendous potential health effects around the world. Thus politics continues to influence infectious disease control on micro and macro levels.

## Ideology and Science

Early in the HIV/AIDS epidemic, the ideologies of scientists, clinicians, and politicians worked against one another as they affected decisions about paying attention to a new and emerging disease. These decisions and the ideology inherent in them were intertwined with beliefs about sexuality and sexual health. The challenge continues today as ideologic and political entities criticize the National Institutes of Health for research funding decisions, not on the basis of scientific merit, but because these groups and persons find research about commercial sex workers, truck drivers, and sexually transmitted diseases to be inappropriate as public health research topics (*12*). In this example, ideology pushes political action to question science and compromise public health.

In each of the cases so far described, both politics and ideology have come into play, and when ideology clouds scientific and public health judgment, decisions go awry and politics become dangerous. Having an ideology or even shouting it from rooftops is perfectly appropriate. One of the fundamental freedoms in our country is the right to believe what we want and express it. But when a person's beliefs bring about public policies that hurt people, they should be held accountable. Condoms and abstinence are well-established, effective means of birth control and STD prevention (*13*). Both have flaws in practical application. Both can be tools in our pursuit of improved health. The denigration of either practice suggests a preference for ideology over science.

Scientists and public health professionals often offer opinions on policy and political issues, and politicians offer theirs on public health policies, sometimes with the support of evidence. This interaction is appropriate and healthy, and valuable insights can be acquired by these cross-discussions. Nevertheless the interaction provides an opportunity for inappropriate and self-serving commentary, for public grandstanding, and for promoting public anxiety for partisan political purposes. Public health professionals should work with politicians to resist ideologic influence, to demand good science, and to make wise decisions and policies.

## Conclusion

For scientists focused exclusively on winning at "NIH bingo," accumulating R01s, KO1s, K15s, RO3s, R13s, and R21s, the interplay between science and politics may be irrelevant. However, most public health scientists and practitioners want to see their efforts improve the public's health. At the same time, scientists require an environment that permits them to work as efficiently and objectively as possible.

**Figure 1:**
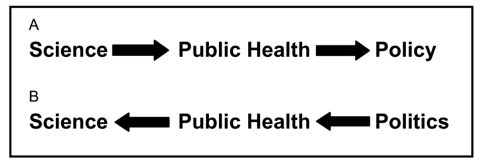
Proper (A) and improper (B) pathways of developing public health policy.

The issue can be succinctly addressed with a simple diagram (Figure 1). On the left is science, essential to inform the practice of public health. In the middle is public health, where science is interpreted and appropriate responses are developed. And on the right is political will and policies necessary to carry out the public health impetus. The tendency is to struggle against the intrusion of politics when it is counter to our own opinion, ignores or misinterprets the science, or is driven by ideology beyond politics as usual. We are right to raise our voices against the intrusion of politics into public health in the second and third circumstances, but should take care in the first one.

The diagram has a clear direction of flow. Science informs public health, which leads to political change. This approach is appropriate and effective to improve health, but the process should only flow in one direction. Reversing directions in public health decision-making is just as hazardous as it is in sewage lines. Even more insidious can be the intrusion of ideology into the process, attempting to reverse the current of the science, public health, politics stream. We have seen cases where ideology or political considerations determine a desirable policy and then seek scientific justification for it, often employing faulty science. When this happens, ideology can diminish the field, discredit the discipline and its practitioners, and undermine what scientists do.

How should infectious disease scientists handle political and ideologic pressures in their own work? One way to handle these pressures is to be connected to the rest of the public health community. Every area of public health faces the same issues: a similar commentary would apply to chronic disease or environmental health. Science and politics are intertwined in myriad ways, and ideologic influences are encountered everywhere. Tremendous concern exists in the United States about infectious diseases. Infectious diseases research no doubt gained the spotlight, and accompanying resources, after the events of September 11, 2001, and the anthrax attacks later that year. But political winds change quickly, and this focus could easily shift.

The infectious disease community needs to see their role within the larger public health context and work actively to forge alliances and collaborations between their work and the work of others. The diagram can continue to flow in the right direction, science to public health to policy, but maintaining this direction requires work, which can be accomplished by recognizing interconnectedness and using the political system to improve public health through good science. Several concrete ways to accomplish the goal exist: 1) Be an advocate for infectious disease control, not just emerging infectious diseases or bioterrorism. 2) Be an advocate for public health, not just infectious diseases. 3) Be an advocate for wise public policy based on science in the context of broader societal considerations. 4) Respect the value of the interplay of science, public health, and politics, but recognize any reversal of flow and resist it when it occurs. We all need to be strong advocates for good science, good public health, and good policies and the positive value that politics can provide for all three of these.
